# MAPK involvement in cytokine production in response to *Corynebacterium pseudotuberculosis* infection

**DOI:** 10.1186/s12866-014-0230-6

**Published:** 2014-09-02

**Authors:** Andréia Pacheco de Souza, Vera Lúcia Costa Vale, Marcos da Costa Silva, Inara Barbosa de Oliveira Araújo, Soraya Castro Trindade, Lília Ferreira de Moura-Costa, Gabriele Costa Rodrigues, Tatiane Santana Sales, Heidiane Alves dos Santos, Paulo Cirino de Carvalho-Filho, Milton Galdino de Oliveira-Neto, Robert Eduard Schaer, Roberto Meyer

**Affiliations:** Biointeraction Department - Immunology and Molecular Biology Laboratory – Health Sciences Institute (ICS), Federal University of Bahia (UFBA), Av. Reitor Miguel Calmon, s/n; Vale do Canela, Salvador, Bahia CEP 40040-040 Brazil; Department of Exact and Earth Sciences (DCET), State University of Bahia (UNEB), Alagoinhas, Bahia Brazil

**Keywords:** *Corynebacterium pseudotuberculosis*, Cytokines, MAPkinases

## Abstract

**Background:**

Caseous lymphadenitis (CL) is a contagious infectious disease of small ruminants caused by *Corynebacterium pseudotuberculosis*. Is characterized by the formation of abscesses in the lymph nodes and intestines of infected animals, induced by inflammatory cytokines. The production of cytokines, such as IL-10, TNF-α, IL-4 and IFN-γ, is regulated by mitogen-activated protein kinase (MAPK) pathway activation. The present study investigated the involvement of MAPK pathways (MAPK p38, ERK 1 and ERK 2) with respect to the production of cytokines induced by antigens secreted by *C. pseudotuberculosis* over a 60-day course of infection. CBA mice (n = 25) were divided into three groups and infected with 10^2^ colony forming units (CFU) of attenuated strain T1, 10^2^ CFU of virulent strain VD57 or sterile saline solution and euthanized after 30 or 60 days. Murine splenocytes were treated with specific inhibitors (MAPK p38 inhibitor, ERK 1/2 inhibitor or ERK 2 inhibitor) and cultured with secreted antigens obtained from pathogenic bacteria (SeT1 or SeVD57).

**Results:**

The MAPK pathways evaluated were observed to be involved in the production of IL-10, under stimulation by secreted antigens, while the MAPK p38 and ERK 1 pathways were shown to be primarily involved in TNF-α production. By contrast, no involvement of the MAPK p38 and ERK 1 and 2 pathways was observed in IFN-γ production, while the ERK 2 pathway demonstrated involvement in IL-4 production only in the mouse splenocytes infected with VD57 under stimulation by SeT1.

**Conclusion:**

The authors hypothesize that MAPK p38 and ERK 1 pathways with respect to TNF-α production, as well as the MAPK p38 and ERK 1 and 2 pathways in relation to IL-10 production under infection by *C. pseudotuberculosis* are important regulators of cellular response. Additionally, the lack of the MAPK p38 and ERK 1/2 pathways in IFN-γ production in infected CBA murine cells stimulated with the two secreted/excreted antigens, in IL-4 production showing involvement only via the ERK 2 pathway under stimulation by SeT1 antigen during 60-day infection period with the virulent strain, suggests that these pathways regulated the production of pro-inflammatory and regulatory cytokines in the splenic cells of CBA mice.

## Background

*Corynebacterium pseudotuberculosis* is a minuscule, Gram-positive, pleomorphic, non-sporulating facultative anaerobic bacillus. Belonging to the genus C*orynebacteriaceae* (*Actinomycetae),* which includes the genera *Rhodococcus* and *Mycobacterium,* it is known to be a causative agent of caseous lymphadenitis (CL) in small ruminants, such as goats and sheep [1,2].

CL is a chronic infectious disease characterized by the formation of granulomas. Infection primarily involves the skin and affected mucosa, followed by the spread of either free bacteria or those inside phagocytes, leading to localized infection in the lymph nodes or internal organs [3].

Once successfully established within the host, chronic infection may persist throughout most, or even the entire lifespan of the animal [4]. This disorder compromises the animal’s skin, skeleton and internal organs, in addition to reducing wool production, limiting weight gain and reproductive efficiency, resulting in reduced birth rates of offspring [5,6] and severe economic hardship for livestock producers, especially small farmers.

The adaptive resistance to infection caused by facultative intracellular bacteria, such as *C. pseudotuberculosis,* is related to CD4 T cells and, more specifically, to clones that produce Th1-type cytokines, mainly IFN-γ and TNF-α. These pro-inflammatory cytokines increase the bactericidal activity of macrophages and activate CD8 T lymphocytes [7,8].

One of the main characteristics of pathogenic mycobacteria is its capability to tolerate and manipulate host immune response, thereby promoting intracellular pathogen survival [9,10]. Initial interactions between macrophages and mycobacteria result in the activation of the intracellular signaling pathway, whereby events mediated by receptors are associated with transcriptional responses and protein translation [11].

The mitogen-activated protein kinases (MAPK) are a subfamily of serine/threonine-specific protein kinases. MAPKs are expressed by all cell types and respond to extracellular stimuli (mitogens) that mediate signal transduction from cell surface receptors to nuclei [12,13]. MAPKs, part of a phosphorylation system in which three kinases are sequentially activated [14,15], are expressed in mammalian cells and translate signals in response to growth factors, pro-inflammatory cytokines and stress conditions.

Three subfamilies of MAPKs have been well-characterized: MAPK p38, which contains four isoforms (α, β, γ, and δ); ERK, the kinase regulated by extracellular signaling, with isoforms p44 (ERK1) and p42 (ERK2); and the protein kinase c-jun N-terminal, with isoforms, JNK 1, JNK 2 and JNK 3 [16,17].

MAPK p38 regulates the expression of several cytokines, is activated in immune cells by inflammatory cytokines, and plays an important role in the activation of host immune response [18]. ERK 1 and 2 are widely expressed and involved in the regulation of meiosis, mitosis and post-mitotic functions in a variety of cells. Cytokines are among the many different stimuli capable of activating the ERK 1 and ERK 2 pathways [19,20]. In addition, MAPK p38 shares about 50% of its homology with ERK [21], suggesting that these two pathways may play similar roles during host immune response.

The present study employed CBA mouse splenocytes to evaluate the potential involvement of the mitogen-activated protein kinases MAPK p38 and ERK1 and 2 with respect to selected *in vitro* cytokine production under stimulation by antigens secreted/excreted by *C. pseudotuberculosis*.

## Results and discussion

### Action of mitogen-activated protein kinase specific inhibitors on the production of TNF-α under stimulation by *C. pseudotuberculosis* antigens

**Figure 1:**
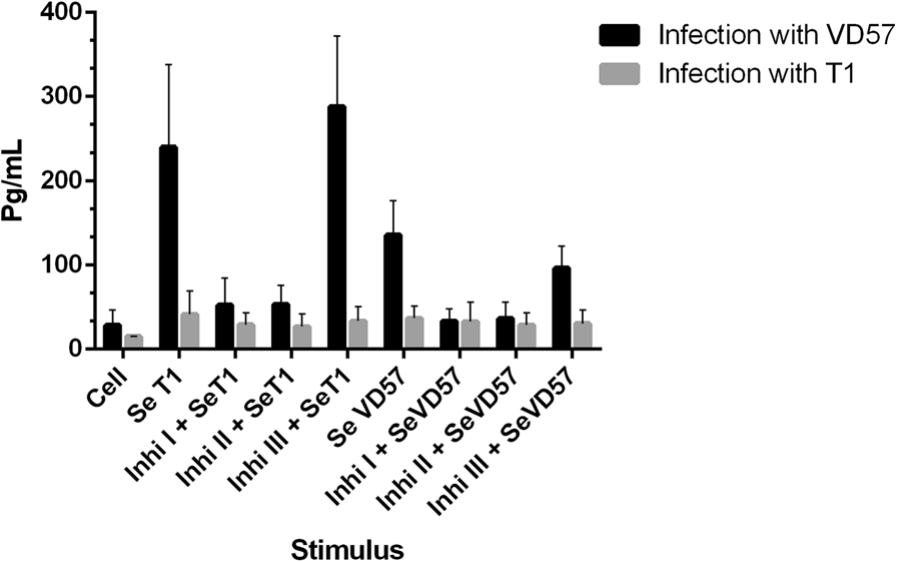
**Effect of MAPK pathway inhibitors MAPK p38 (Ini I), ERK 1 / 2 (Ini II) and ERK 2 (Ini III) on the production of TNF-α by splenic cells obtained from CBA mice after 30 days of infection with virulent (VD57) and attenuated (T1)** ***C. pseudotuberculosis*** **strains, under stimulation by secreted/excreted antigens (SeT1 and SeVD57).** Each experiment was carried out ≥3 times in triplicate. *p < 0.05 with respect to stimulation by SeT1; # *p* < 0.05 with respect to stimulation by SeVD57.

An analysis of the group of CBA mice infected with the virulent VD57 strain of *C. pseudotuberculosis* for 30 days revealed a statistically significant inhibition (P = 0.016) of TNF-α production. This was induced only by the MAPK p38 (Inhib 1) and ERK 1/2 (Inhib 2) inhibitors under stimulation by both virulent and attenuated secreted/excreted antigens. However, also at 30 days after infection, cells obtained from the group of animals infected with the attenuated T1 strain showed no statistically significant inhibition in TNF-α production following treatment with MAPK inhibitors under stimulation by secreted antigens (Figure 1).

**Figure 2:**
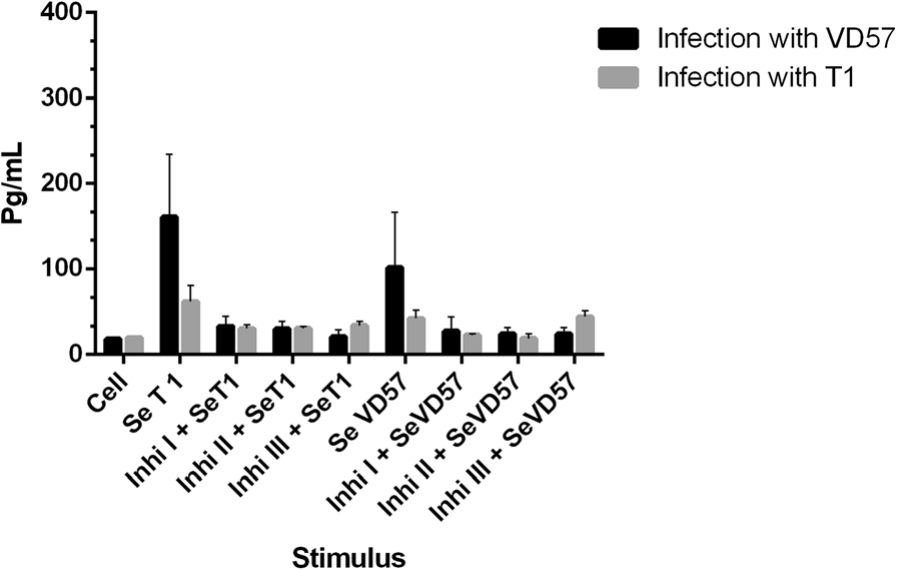
**Effect of MAPK pathway inhibitors MAPK p38 (Ini I), ERK 1 / 2 (Ini II) and ERK 2 (Inb III) on the production of TNF-α by splenic cells obtained from CBA mice after 60 days of infection with virulent (VD57) and attenuated (T1)** ***C. pseudotuberculosis*** **strains, under stimulation by secreted/excreted antigens (SeT1 and SeVD57).** Each experiment was carried out ≥3 times in triplicate. *p < 0.05 with respect to stimulation by SeT1; # *p* < 0.05 with respect to stimulation by SeVD57.

In the group of mice infected with the virulent strain for 60 days, a statistically significant inhibition (P = 0.009) in TNF-α production was observed in the splenic cultures treated with each of the three MAPK inhibitors under stimulation by SeT1. However, in the treated cells that were stimulated with SeVD57, a statistically significant decrease (P = 0.009) in TNF-α was observed only in those cultures treated with the ERK 1/2 inhibitor. In the group of mice infected with the attenuated strain for 60 days, statistically significant inhibition (P = 0.009) of TNF-α was also observed in cultures treated with any one of the three inhibitors under stimulation with SeT1. By contrast, under stimulation by SeVD57, a statistically significant reduction (P = 0.009) in TNF-α production was observed only in the groups treated with the MAPK p38 and ERK1/2 inhibitors (Figure 2).

### Action of mitogen-activated protein kinase specific inhibitors on the production of IFN-γ under stimulation by *C. pseudotuberculosis* antigens

**Figure 3:**
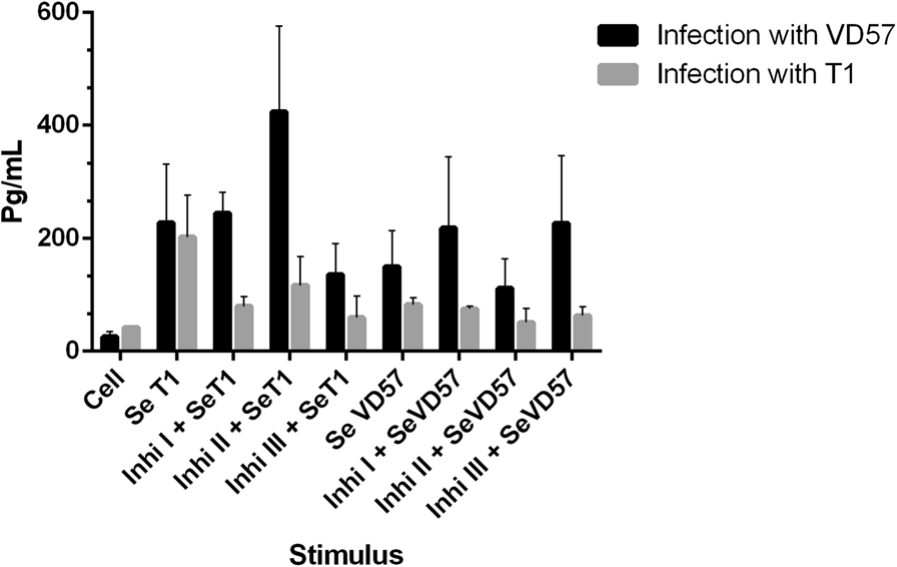
**Effect of MAPK pathway inhibitors MAPK p38 (Ini I), ERK 1 / 2 (Ini II) and ERK 2 (Inb III) on the production of IFN-γ by splenic cells obtained from CBA mice after 30 days of infection with virulent (VD57) and attenuated (T1)** ***C. pseudotuberculosis*** **strains, under stimulation by secreted/excreted antigens (SeT1 and SeVD57).** Each experiment was carried out ≥3 times in triplicate.

After 30 days of infection with either the virulent or attenuated strain of *C. pseudotuberculosis*, no statistically significant inhibition of IFN-γ production was observed in CBA cells treated with any of the three MAPK inhibitors and subsequently stimulated with secreted antigens from either strain (Figure 3).

**Figure 4:**
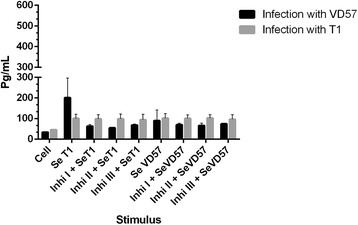
**Effect of MAPK pathway inhibitors MAPK p38 (Ini I), ERK 1 / 2 (Ini II) and ERK 2 (Inb III) on the production of IFN-γ by splenic cells obtained from CBA mice after 60 days of infection with virulent (VD57) and attenuated (T1)** ***C. pseudotuberculosis*** **strains, under stimulation by secreted/excreted antigens (SeT1 and SeVD57).** Each experiment was carried out ≥3 times in triplicate.

Similar results were obtained after 60 days of infection with both the virulent and attenuated strains, as no statistically significant inhibition of IFN-γ was observed in the cells treated with any of the three MAPK inhibitors followed by stimulation with secreted antigens (Figure 4).

### Action of mitogen-activated protein kinase specific inhibitors on the production of IL-10 under stimulation by *C. pseudotuberculosis* antigens

**Figure 5:**
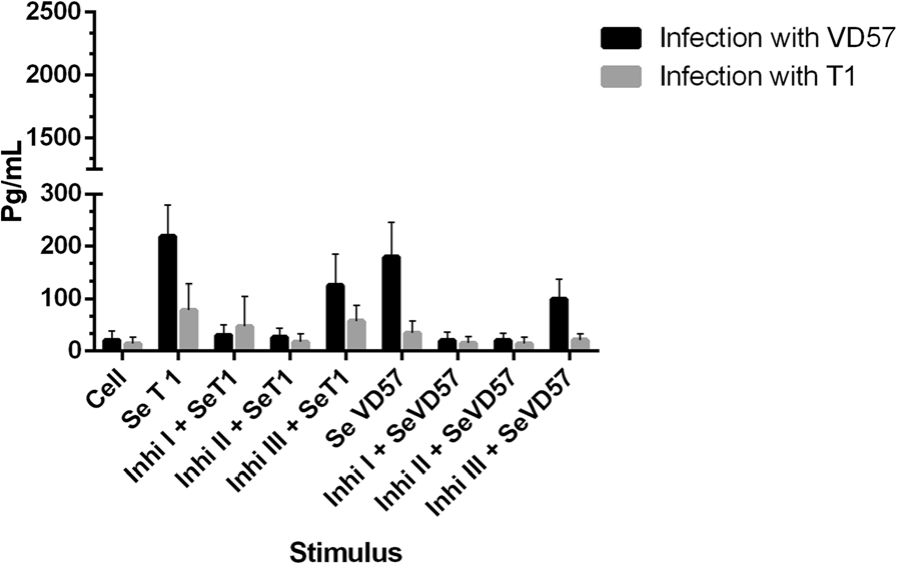
**Effect of MAPK pathway inhibitors MAPK p38 (Ini I), ERK 1 / 2 (Ini II) and ERK 2 (Inb III) on the production of IL-10 by splenic cells obtained from CBA mice after 30 days of infection with virulent (VD57) and attenuated (T1)** ***C. pseudotuberculosis*** **strains, under stimulation by secreted/excreted antigens (SeT1 and SeVD57).** Each experiment was carried out ≥3 times in triplicate. *p < 0.05 with respect to stimulation by SeT1; # *p* < 0.05 with respect to stimulation by SeVD57.

After 30 days of infection with the virulent VD57 strain, a statistically significant decrease (P = 0.008) was observed in IL-10 production by splenic cells pretreated with any one of the three MAPK inhibitors and then subsequently stimulated with the SeT1 antigen. When stimulated with SeVD57, a statistically significant reduction in IL-10 (P = 0.009) was observed when the MAPK p38 and ERK 1/2 pathways were inhibited. Mice infected for 30 days with the attenuated T1 strain showed a significant reduction (P = 0.028) in IL-10 production only when the ERK 1/2 inhibitor was applied in conjunction with stimulation by SeT1 (Figure 5).

**Figure 6:**
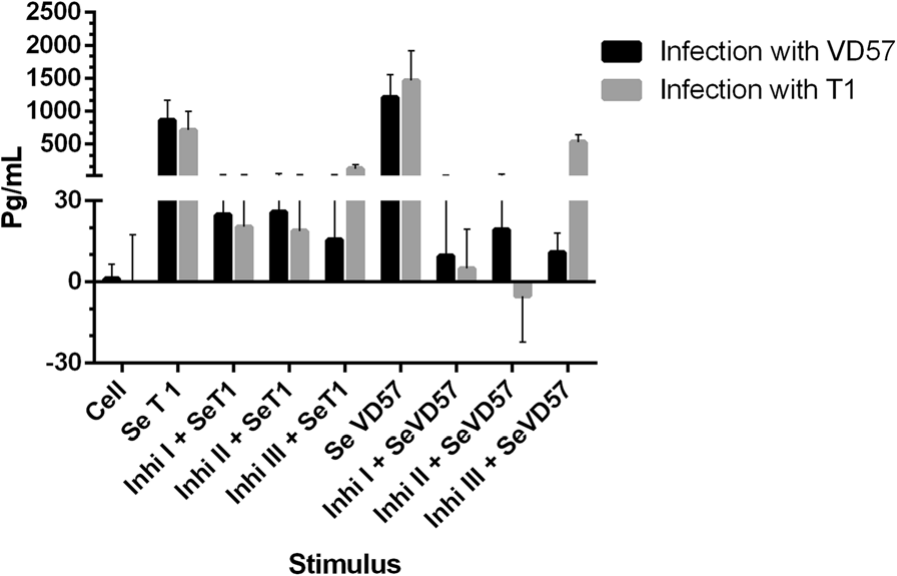
**Effect of MAPK pathway inhibitors MAPK p38 (Ini I), ERK 1 / 2 (Ini II) and ERK 2 (Inb III) on the production of IL-10 by splenic cells obtained from CBA mice after 60 days of infection with virulent (VD57) and attenuated (T1)** ***C. pseudotuberculosis*** **strains, under stimulation by secreted/excreted antigens (SeT1 and SeVD57).** Each experiment was carried out ≥3 times in triplicate. *p < 0.05 with respect to stimulation by SeT1; # *p* < 0.05 with respect to stimulation by SeVD57.

At 60 days after infection with both virulent and attenuated strains, all three MAPK inhibitors induced statistically significant decreases (P = 0.009) in IL-10 production under stimulation by secreted/excreted antigens. The only exception was cells infected with the attenuated strain, which were treated with ERK 2 inhibitor and stimulated by SeVD57 (Figure 6).

### Action of mitogen-activated protein kinase specific inhibitors on the production of IL-4 under stimulation by *C. pseudotuberculosis* antigens

**Figure 7:**
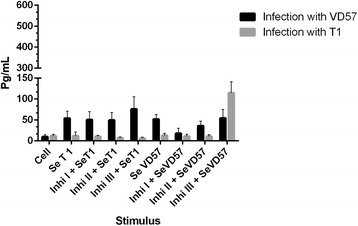
**Effect of MAPK pathway inhibitors MAPK p38 (Ini I), ERK 1 / 2 (Ini II) and ERK 2 (Inb III) on the production of IL-4 by splenic cells obtained from CBA mice after 30 days of infection with virulent (VD57) and attenuated (T1)** ***C. pseudotuberculosis*** **strains, under stimulation by secreted/excreted antigens (SeT1 and SeVD57).** Each experiment was carried out ≥3 times in triplicate.

**Figure 8:**
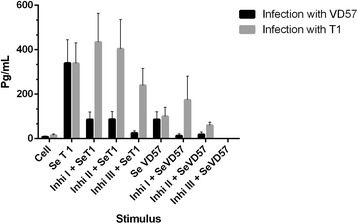
**Effect of MAPK pathway inhibitors MAPK p38 (Ini I), ERK 1 / 2 (Ini II) and ERK 2 (Inb III) on the production of IL-4 by splenic cells obtained from CBA mice after 60 days of infection with virulent (VD57) and attenuated (T1)** ***C. pseudotuberculosis*** **strains, under stimulation by secreted/excreted antigens (SeT1 and SeVD57).** Each experiment was carried out ≥3 times in triplicate. *p < 0.05 with respect to stimulation by SeT1.

After 30 days of infection with both the virulent and attenuated strains, no statistically significant decreases in IL-4 production were observed in any cell cultures treated with MAPK inhibitors under stimulation by either SeT1 or SeVD57 antigens (Figure 7). Interestingly, after 60 days of infection, a statistically significant inhibition (P = 0.047) was detected in cells infected with the virulent VD57 strain and treated with the ERK 2 inhibitor under stimulation by SeT1 (Figure 8).

The data presented herein strongly suggest that, under the experimental protocol described above, the main pathways involved in the production of TNF-α are MAPK p38 and ERK 1. In addition, the present study also demonstrated the involvement of the MAPK p38 and ERK 1 and 2 pathways with respect to the production of IL-10. Recent studies have suggested that the MAPK signaling pathway is essential to the production of pro-inflammatory cytokines, such as TNF-α and IL-10 [22,23]. The activation of the MAPKs pathway varies in terms frequency and extent in accordance with pathogen species and degree of virulence [24,25].

After infection for 30 days with the virulent VD57 strain, the data herein suggest that the MAPK p38 and ERK 1 pathways are involved in the production of TNF-α when stimulated by secreted/excreted antigens. By contrast, none of the MAPKs pathways appeared to be involved in TNF-α production after 30 days of infection by the attenuated T1 strain. Nonetheless, after 60 days of infection under stimulation by the SeT1 antigen, all three analyzed MAPK pathways demonstrated involvement in TNF-α production. Furthermore, in cell cultures stimulated with the SeVD57 antigen for 60 days, the MAPK p38 and ERK 1 pathways demonstrated involvement in cytokine production, while the ERK 2 pathway did not.

A previous study conducted by Sim et al. [26] found that *in vitro* infection by *M. avium* in the monocytes of healthy patients was able to successfully activate the MAPK p38 and ERK 1/2 pathways and positively regulate TNF-α production. However, this same study also employed monocytes from diseased patients with *M. abscessus* and found that *in vitro* infection with *M. avium* decreased the expression of MAPK p38 and ERK 1/2, in addition to reducing TNF-α production.

Basler et al. [27] investigated the production of TNF-α by murine macrophages infected with *M. avium* subspecies *paratuberculosis* (pathogenic) and *M. smegmatis* (non-pathogenic) using MAPK p38 (SB203580) inhibitors. These authors found that the MAPK p38 pathway was indeed involved in TNF-α production, with low levels observed under infection by the pathogenic species. According to their findings, the induced decrease in TNF-α by pathogenic mycobacteria was associated with a reduction in the duration and extent of MAPK p38 phosphorylation.

Other studies have demonstrated the importance of the pro-inflammatory cytokine TNF-α, most notably at the site of infection [28,29]. As such, the blocking of TNF-α by monoclonal antibodies inhibits the formation of well-differentiated granulomas in experimental infection models employing *M. tuberculosis*, resulting in the dissemination of the pathogen [30,31].

According to Rocco and Irane [32], the interference of intracellular signaling is fundamental to the strategy of virulence of intracellular pathogens, such as *M. tuberculosis* and *M. avium.*

The aforementioned study by Sim et al. [26] reported on the importance of the activation of the MAPK p38 and ERK 1/2 pathways with respect to TNF-α production. These authors demonstrated that the insufficient activation of the MAPK p38 pathway in response to pathogenic mycobacteria may represent an important mechanism, due to the negative regulation of host immune response, thus allowing the pathogen to persist intracellularly.

With respect to the involvement of the three evaluated MAPK pathways in the production of IL-10, the findings presented herein are in partial agreement with the data presented by Méndez-Samperio et al. [33]. Theses authors treated human lung epithelial cells with the SB203580 MAPK p38 inhibitor, demonstrating the involvement of MAPK p38 signaling in the secretion of IL-10 following infection with *M. bovis* BCG cells. However, this same study also used PD98059 to inhibit the MEK (MAPK / ERK) pathway and found that this pathway was not involved in IL-10 production, which is known to be responsible for the phosphorylation and subsequent activation of ERK.

According to Souza et al. [11], blocking the MAPK p38 pathway with SB203580 prior to infecting bovine monocytes with *M. avium* subspecies *paratuberculosis* resulted in decreased IL-10 expression. Elevated IL-12 expression was also observed along with increased acidification of phagocytes and a reinforcement of parasite killing, suggesting that the overproduction of IL-10 mediated by the MAPK p38 pathway may play an important role in the mitigation of antimicrobial activity by phagocytes. This association constitutes a key mechanism involved in the capability of *M. avium* subsp*. paratuberculosis* to survive in bovine monocytes.

IL-10 is known to inhibit the transcription and production of pro-inflammatory cytokines, such as TNF-α and IL-12, as well as to impede antigen presentation and inhibit the proliferation of T cells that produce IFN-γ [34]; as such, this cytokine clearly suppresses the Th1 response.

Taken together, the findings presented in previous studies indicate that the early activation of the MAPK p38 pathway may be a common mechanism by which virulent mycobacterial organisms suppress the antimicrobial response of susceptible host cells, allowing pathogens to persist intracellularly.

The activation of the MAPK p38 signaling pathway leads to rapid IL-10 expression under infection by intracellular bacteria, which in turn attenuates antimycobacterial activity, including the production of proinflammatory cytokines and phagosome maturation. Thus, it is possible that further manipulation of the MAPK p38 pathway may lead to a better understanding of the escape mechanisms used by *C. pseudotuberculosis* to evade host immune response during infection*.*

The data presented herein suggest that none of the MAPK pathways studied were involved in the production of IFN-γ. This finding is unsurprising, as the literature contains various studies showing the involvement of MAPK p38 and ERK 1/2 in the production of pro-inflammatory and/or regulatory cytokines in experimental models that employ a variety of intracellular parasites [25,35,36].

IL-4 production was observed to involve only the ERK 2 pathway in CBA mice after 60 days of infection with the virulent strain under stimulation by the SeT1 antigen. This finding suggests the differential involvement of a cascade of cellular signaling in response to virulent and attenuated *C. pseudotuberculosis* at various infection times. These differences in pathway activation may result from the differential expression of molecular compounds by the two strains considered herein, which are then recognized by macrophage receptors.

## Conclusion

The present study serves to contribute to the working body of knowledge on the interaction of *C. psedotuberculosis* and immune resonse of the host, as it clearly demonstrates the importance of the MAPK p38 and ERK 1 pathways with respect to TNF-α production, as well as the MAPK p38 and ERK 1 and 2 pathways in relation to IL-10 production under infection by *C. pseudotuberculosis*, providing evidence that the respective signaling pathways are important regulators of cellular response to this pathogen.

Additionally, the lack of involvement of the MAPK p38 and ERK 1/2 pathways in IFN-γ production in infected CBA murine cells stimulated with the two secreted/excreted antigens, considered in conjunction with the production of IL-4 showing involvement only via the ERK 2 pathway under stimulation by SeT1 antigen during 60-day infection period with the virulent strain, suggests that these signaling pathways may cooperate to regulate the production of only pro-inflammatory and regulatory cytokines in the splenic cells of CBA mice. Further studies should be conducted to validate this hypothetical assumption.

## Methods

### *C. pseudotuberculosis* strain selection

The present study employed attenuated and virulent strains of *C. pseudotuberculosis*, T1 and VD57, respectively. Both of these were isolated from the granuloma of goats taken from rural regions of the state of Bahia, located in northeastern Brazil, and then stored in the Department of Microbiology collection center at the Health Sciences Institute of the Federal University of Bahia (ICS-UFBA).

The strain T1, had its identification confirmed by Gram staining, colony morphology, synergistic hemolytic activity with CAMP factor of *Rhodococcus equi*, urease and catalase production. A commercial kit to perform a more reliable identification was also used (API Coryne - bioMérieux). According to previous studies, this strain can’t induce disease in goats [37,38] and mice [8], and was used as a vaccinal strain [39]. Furthermore, during the synergistic hemolysis test, we observed a pattern of less severe hemolysis when compared with other wild strains.

The pathogenic strain, employed as a challenge to vaccinated animals, was named VD57, and had a similar identification process as the attenuated strain. This strain induces caseous lymphadenitis in goats [38–40] and mice [8]. The bacteria were grown in the ICS-UFBA microbiology department laboratory in Brain Heart Infusion (BHI) broth. Then the strains T1 and VD57 they were cultured for 72 hours at 37°C to obtain secreted antigens (SeT1 and SeVD57, respectively). Cultures were also later used to infect experimental animals.

### Obtainment of secreted/excreted antigens

Bacterial culture supernatants were collected following centrifugation at 12,000 xg for 30 minutes at 4°C. Bacteria-free supernatant was filtered using a 0.2 μm membrane, followed by the gradual addition of 30% ammonium sulfate (176 g/liter). pH values were measured immediately thereafter and adjusted as necessary to 4.0 using concentrated HCl. Next, n-butanol was added in an identical proportion to the volume of obtained supernatant. Following vortex agitation for one minute and one hour of rest at room temperature (24°C), the protein fraction was precipitated at the interface and collected, then resuspended in PBS and centrifuged at 1350 xg for 10 minutes. Dialysis was subsequently performed on the obtained media in a diluted 3 M phosphate buffer (ratio: 20 mL of buffer to 1 L of distilled water) using a cold chamber for 48 hours. The obtained antigen protein concentration was determined using the *Lowry* method in accordance with manufacturer guidelines (Bio-Rad, Hercules, CA).

### Animals and experimental infection protocol

Twenty-five 60-day-old male and female CBA mice were supplied by the Gonçalo Moniz Research Center (FIOCRUZ - BA) animal care facility. All animals were kept in cages in a pathogen-free environment under controlled temperature, humidity and light conditions, and were fed a balanced diet of rations and distilled water *ad libitum*. The experimental infection protocol entailed the intraperitoneal injection of 10 animals with 10^2^ colony forming units (CFU) of T1 *C. pseudotuberculosis* and 10 animals with 10^2^ CFU of the VD57 strain, while five control mice received sterile saline injections. After 30 days, five of each mouse group infected with either T1 or VD57 were sacrificed to assess cytokine production *in vitro*, and this same procedure was repeated at 60 days with the remaining animals. The present study was approved by the Institutional Animal Care and Use Committee of the Federal University of Bahia (Protocol number 006/2010).

### Splenocyte collection and culturing

Infected mouse spleens were aseptically removed and washed twice in RPMI 1640 medium (Sigma). Spleen cells were collected by divulsion and placed in RPMI medium (3 mL) supplemented with 10% fetal bovine serum and 1% antibiotic (penicillin and streptomycin). Obtained cells were diluted at 1:100 (30 μl of acetic acid, 960 μl of trypan blue stain and 10 μl of cells) and counted in a Neubauer chamber to determine cellular viability as well as to adjust concentration levels. Splenic cells were then cultured in 24-well plates at a final concentration of 10^6^ cells per mL.

### Treatment of splenocytes with MAPK p38 and ERK 1/2 inhibitors

Splenic cell cultures were exposed to the following specific inhibitors (Biosource/Life Technologies, Grand Island, USA): 1) SB203580 (MAPK p38 inhibitor); 2) U0126 (ERK 1/2 inhibitor) or 3) ERK activation inhibitor peptide I (ERK 2 inhibitor) at a concentration of 40nM for one hour before stimulation with secreted antigens (SeT1 or SeVD57).

### Splenocyte stimulation using secreted/excreted antigens

One of the following two *C. pseudotuberculosis* antigens were added to the plate wells*:* SeT1 (attenuated strain secretion) or SeVD57 (wild-type strain secretion) at a concentration of 40 μg/mL. Pokeweed mitogen (PWM) was used at a concentration of 2.5 μg/mL for positive controls, while non-stimulated cells (antigens) were used as negative controls.

Following stimulation, all well plates were incubated for 120 hours (at 37°C and 5% CO_2_), then collected. After centrifugation, the culture supernatants were stored at −20°C until the time of cytokine quantification.

### Cytokine quantification

The levels of cytokines TNF-α, IFN-γ, IL10 and IL-4 were measured in the culture supernatants by the “sandwich” ELISA technique using commercial kits from R&D (Minneapolis, USA) in accordance with manufacturer guidelines as summarized below:

Plates (Costar/Corning, Tewksbury, USA) were sensitized with 100 μL/well of capture antibody diluted in filtered PBS and incubated overnight at room temperature (24°C). The plates were then washed ten times with PBS-T (this same washing procedure was used repeatedly at each stage). Blocking was performed with 2% PBS-BSA for 2 h at room temperature (this same incubation procedure was employed at all stages). Next, the plates were washed, 100 μL of samples, control or standard dilutions per well (diluted in 1% PBS-BSA) at concentrations of 2000 pg/mL, 1000 pg/mL, 500 pg/mL, 250 pg/mL, 125 pg/mL, 62.5 pg/mL, 31.2 pg/mL for IFN-γ, IL10 and TNF-α; while for IL-4 the following concentrations were used: 1000 pg/mL, 500 pg/mL, 250 pg/mL, 125 pg/mL, 62.5 pg/mL 31.2 pg/mL, 15.6 pg/mL. Next, all plates were incubated for 2 h and immediately washed, followed by the addition of a detection antibody diluted in 1% PBS-BSA (100 μL/well) and an additional incubation period of 2 h. After washing, conjugated streptavidin-HRP was diluted at 1:200 and then added to each well (100 μl), followed by a 20-minute incubation period. Immediately thereafter, all plates were washed again, tetramethylbenzidine (TMB) liquid substrate was added (100 μL per well), followed by a subsequent incubation period of 20 minutes in a light-free environment. Next, 50 μL of stop solution (2 N H_2_SO_4_) was added to each well and photocolorimetric readings were taken using 450 to 620 nm filters in a microplate reader (Bio-Rad, Hercules, CA).

### Statistical analysis

All data from two experimental infection groups and five control mice were analyzed using SPSS (Statistical Package for Social Sciences) version 9.0 for Windows. The nonparametric Kruskal-Wallis test was used to compare means among groups, while the Mann–Whitney *U*-test was employed to make pairwise comparisons among independent groups. Results were considered statistically significant when *p* < 0.05.

## Abbreviations

MAPK: Mitogen-activated protein kinase
CL: Caseous lymphadenitis
CFU: Colony forming units
ERK: Kinase regulated by extracellular signaling
ELISA: Enzyme linked immuno sorbent assay
TNF-α: Tumor necrosis factor alpha
IFN-γ: Interferon gamma
IL-10: Interleukin-10
IL-12: Interleukin-12
IL-4: Interleukin-4
TMB: Tetramethylbenzidine
PBS: Phosphate buffered saline
BSA: Bovine serum albumin
SPSS: Statistical package for social sciences.
